# Potential smoke-free dividend across local areas in England: a cross-sectional analysis

**DOI:** 10.1136/tc-2023-058264

**Published:** 2024-03-20

**Authors:** Damon Morris, Duncan Gillespie, Martin J Dockrell, Mark Cook, Marie Horton, Jamie Brown, Tessa Elisabeth Langley

**Affiliations:** 1 Sheffield Addictions Research Group, School of Medicine and Population Health, The University of Sheffield, Sheffield, UK; 2 SPECTRUM Consortium, UK; 3 Office for Health Improvement and Disparities, London, UK; 4 Behavioural Science and Health, University College London, London, UK; 5 Nottingham Centre for Public Health and Epidemiology, University of Nottingham, Nottingham, UK

**Keywords:** Economics, Illegal tobacco products, Cessation, Disparities, End game

## Abstract

**Background:**

The value that might be added to local economies each year through the money that people who smoke tobacco would save if everyone quit smoking is called the ‘smoke-free dividend’. This study aimed to estimate the value of the smoke-free dividend across local areas in England, and how it relates to the average income in those areas.

**Methods:**

The study was a cross-sectional descriptive analysis of tobacco expenditure from the Smoking Toolkit Study (STS) matched to income and smoking prevalence data for English local authorities. The STS sample was from 2014 to 2020 and comprised 18 721 adults who smoke cigarettes. Self-reported expenditure estimates from the STS were adjusted for under-reporting. This adjustment aimed to align the total expenditure estimate with figures derived from government tax receipts and national estimates of illicit tobacco use. The smoke-free dividend is calculated as 93% of spending on legal tobacco, which is the percentage estimated to leave the local economy, plus 100% of spending on illicit tobacco.

**Results:**

The total dividend in England is estimated to be £10.9 billion each year, which equates to £1776 per person who smokes or £246 per adult regardless of smoking status. The estimated dividend is greater in areas with lower average income, with a correlation coefficient of −0.521 (95% CI −0.629, –0.392) between the average income of local areas and the dividend per adult.

**Conclusions:**

This study has estimated that local economies could gain a substantial dividend if everybody stopped smoking, which is larger in lower income areas, meaning that geographical economic inequalities could be reduced.

**WHAT IS ALREADY KNOWN ON THIS TOPIC**

- Most spending on tobacco goes to government as tax, to the tobacco industry as profits or as proceeds to the illicit trade. In England, only 7% of spending on tobacco remains within the local economy. There is therefore a large potential economic dividend to people who smoke and local economies if everyone were to stop smoking. The smoke-free dividend for England was previously estimated at £7 billion.

**WHAT THIS STUDY ADDS**

- This study estimates the smoke-free dividend for England using a method to account for under-reporting of spending on tobacco by individuals in survey data. The estimate of the economic dividend is £10.9 billion.

**HOW THIS STUDY MIGHT AFFECT RESEARCH, PRACTICE OR POLICY**

- The smoke-free dividend was estimated to be largest in local areas with the lowest average income. These are also the areas with the highest smoking rates. The potentially large dividend strengthens the case for tobacco control policy action to reduce smoking rates, particularly in more deprived communities. The methods used here can be used to conduct a similar analysis for other countries.

## Introduction

 Almost all the money spent on tobacco flows directly out of local economies. Expenditure on tobacco also places a significant financial burden on people who smoke, especially those with little disposable income, which is in addition to any loss of income and additional healthcare expenditure caused by the health consequences of smoking.^1^ The experience of this financial burden in the population is made more acute because rates of smoking are highest for people in poorer socioeconomic circumstances.^2^ For example, in England in 2020, 9.6% of people in managerial and professional occupations smoked tobacco, compared with 24.5% for people in routine and manual occupations.^3^ Expenditure on tobacco can directly exacerbate poverty by reducing the resources available to spend on other goods and services.^4^,^7^ A UK study found that 230 000 households, comprising 400 000 adults and 180 000 children, fell below the poverty line because of tobacco expenditure.^6^ Another study has estimated that 135 000 adults with a common mental disorder in the UK would be defined as living in poverty if their income were assessed after their expenditure on tobacco had been subtracted from it.^7^ In the face of rapidly rising living costs,^8^ there is an increased urgency to highlight the financial burden that tobacco use places on households and communities, and the potential financial dividend to local economies that quitting smoking might bring.

In 2019, the government set an objective for England to be smoke free (defined as achieving smoking rates of 5% or less) by 2030,^9^ and in 2022 they commissioned an independent report led by Khan that recommended bold new policies to set the country towards making smoking obsolete.^10^ As smoking rates approach the smoke-free target, and then continue to fall to make smoking obsolete (ie, to approach zero), money that would otherwise have been spent on tobacco will be available to people who used to smoke, freeing up household budgets. This additional money might then be used to purchase other goods and services, which are more likely to benefit local economies than money spent on tobacco. The ‘smoke-free dividend’ to the economy stems from the financial redistribution that occurs when people who smoke quit and hence stop spending money on tobacco.^1^ It is specifically the part of tobacco expenditure which currently flows out of the local economy as tax, industry profit and spending on illicit products. When this spending is redistributed to other consumption goods with larger profit margins to local retailers, more of the spending is retained as income locally, rather than leaving the local economy as profit to tobacco corporations or tax to central government.

Estimating the additional money that could go to local economies if everyone stopped smoking requires subtracting the revenue that is already retained by small retailers as profits from tobacco sales. Most of the money spent on tobacco is made up of government taxes, profits to the tobacco industry or transferred to the illicit trade. It is estimated that only 7% of the revenue from legal tobacco sales in Great Britain is retained by small retailers, for whom tobacco might only be one of many products that they sell.^11^ Thus, in the absence of smoking, the potential economic dividend to local economies could be 93% of the total money spent on legal tobacco (ie, the total spend minus the 7% that already goes to retailers). In addition, spending which would have gone to the illicit trade could also go back into local economies.

The only prior estimate of the potential smoke-free divided for England was made in a report published by the Royal College of Physicians (RCP), which estimated that the potential total dividend from quitting smoking in 2018 was £7 billion.^1^ This means that if everyone stopped smoking, then in 1 year up to £7 billion could re-enter the economy. However, this estimate of the dividend is acknowledged in the report to be an underestimate due to individuals under-reporting their tobacco consumption in survey data^12^; tobacco duty receipts alone in that period were £9 billion. The RCP report figure does not attempt to correct for under-reporting, nor does it explicitly address that some spending will be on illicit tobacco. Both of these omissions in the methodology will result in an underestimate of the value of the smoke-free dividend.

This study aimed to conduct the first comprehensive estimate of the potential value of the smoke-free dividend for England, and how this would be distributed across local areas. We define a local area in our analysis as an English upper tier local authority (UTLA). A local authority is an area of responsibility for delivery of public services by local government in England. To accurately estimate the value of the dividend, we included expenditure on both legal and illicit tobacco and adjusted the estimate of tobacco expenditure upwards to account for under-reporting of tobacco consumption in the survey data. To help understand the scale of the dividend and how it might be distributed in the population, we report estimates in terms of: (a) the total dividend for each UTLA; (b) the dividend per capita, that is, the total dividend divided by the number of adults living in each area; and (c) the dividend per person who currently smokes. We then investigated geographical variation in the value of the dividend in relation to the average weekly income of households in each UTLA, where that income is adjusted for factors including family size and housing costs.

## Methods

### Study design

We used self-reported data on tobacco expenditure from the Smoking Toolkit Study (STS),^13^ a repeated cross-sectional study collected from a representative monthly sample of the English adult population, including both people who do and do not smoke aged over 18 years, and local authority identifiers. We pooled STS data from April 2014 to February 2020, covering the period from when the local authority identifiers became available in the data to the start of the COVID-19 pandemic in the UK. The data were pooled over this period to maximise the number of observations available for local authority-level analyses. We also used data on average income by local authority from the Office for National Statistics (ONS).^14^ Throughout the study we use the term ‘tobacco’ to refer to factory-made cigarettes and hand-rolled tobacco. Other tobacco products, for example, cigars, make up a negligible fraction of all tobacco consumed in England and so were excluded from the analysis. The smoke-free dividend was calculated at the local authority level and then summed to produce an aggregate estimate of the dividend at government office region and national level. To correct for under-reporting of expenditures on tobacco in the survey data, we calculated an upshift factor that was applied to the individual-level tobacco expenditure data from the STS (online supplemental file 1). Analysis was conducted without pre-registration.

### Calculation of tobacco expenditure

The key data field from the STS was respondents’ self-reported weekly expenditure on tobacco, which we adjusted to December 2018 prices using the Consumer Price Index for tobacco products.^15^ The ‘price year’ was set to align with the year for which all spending, price, tax and income data used in the analyses were available. The sample was restricted to people who are current smokers of tobacco with non-missing weekly tobacco expenditure data. We assumed that the data on tobacco consumption and expenditure in the STS included both legal and illicit sources of tobacco. Tobacco expenditure from the STS was calculated as the average weekly expenditure on tobacco by people who smoke in each local authority, weighted by the STS survey weights. This expenditure was then multiplied by an estimate of the total number of people who smoke in each local authority, which was obtained from the Local Tobacco Control Profiles for England.^3^ The resulting value of total weekly expenditure on tobacco was then annualised.

To correct the tobacco expenditure estimates from the STS for under-reporting, we derived a reference value for actual tobacco expenditure by adding the expenditure on legal tobacco, implied by government duty receipts, to the expenditure on illicit tobacco implied by estimates of illicit tobacco consumption and the price of illicit tobacco (see online supplemental file 1 for the detail of this calculation and the data sources used). For legal tobacco, price and tax data were used to estimate the proportion of the retail price paid as excise duty, and this proportion was then used to scale the total duty receipts into an estimate of total tobacco expenditure. By calculating the ratio between our reference value for total tobacco expenditure and the total tobacco expenditure estimated from the self-reported data in the STS, we estimated an upshift factor, which we then applied to the individual STS expenditure estimates.

### Calculation of the smoke-free dividend

The smoke-free dividend was calculated as the total upshifted tobacco expenditure minus the 7% of legal expenditure on tobacco that is expected to be retained as profits by local retailers.^11^ We therefore attributed 93% of legal expenditure to the smoke-free dividend, and to this we added 100% of illicit expenditure (which was estimated as 11.4% of total expenditure), that is, *dividend=(0.93×legal expenditure)+illicit expenditure*. Profits of local retailers are excluded from the dividend figure as this is money which is already retained in the local economy.

We used the geographical variation in the data to calculate the smoke-free dividend at broad region and UTLA levels. England consists of 151 UTLAs. We assumed that the division of expenditure between legal and illicit tobacco at the national level applied to each local authority. For each region and local authority, we calculated: (1) the dividend per capita, that is, the total dividend divided by the number of adults aged 18+ in each area, and (2) the dividend per adult who smokes. These two figures are different ways of illustrating the scale of the potential benefits to local economies from people who smoke giving up smoking, by showing the total money per adult and per person who smokes.

### Analysis of variation in the smoke-free dividend

We investigated the geographical variation in the smoke-free dividend in relation to the average income of each local authority area. Average income data were obtained for middle layer super output areas for the financial year ending March 2018^16^ and were aggregated to produce population-weighted average incomes for regions and local authorities. The income measure used is net equivalised household income after considering housing costs, calculated by the ONS using Organisation for Economic Co-operation and Development equivalence scales.^17^ Equivalisation adjusts household income to account for differences in household size and composition to account for differences in the incomes needed by different households to acquire similar standards of living. The average income data were then used to estimate the proportion of income spent on tobacco. Heat maps are used to illustrate the locations of local authorities with the highest dividends per capita, and to facilitate visual comparison to the locations of high and low-average income. Of the 151 UTLAs in the data, 10 had fewer than 10 observations in the STS data sample, and so were excluded from all local authority analysis. We estimated Pearson correlation coefficients (that range from −1 to 1) to describe the associations between the smoke-free dividend, average income, tobacco expenditure, the average number of cigarettes smoked per day and the proportion of income spent on tobacco. The coefficients provide a summary of the direction and strength of the relationships. We show the degree of uncertainty in these relationships using a threshold for significance of 0.05 (two-way) to produce 95% CIs. Where the CIs around a correlation coefficient include zero, this indicates a greater than 5% probability that the observed relationship between the variables is due to chance.

For a complete list of all data sources used in the analysis, see online supplemental file 2. The code and publicly available data underlying the analysis have been made open source.^18 19^

## Results

The STS data sample contained 112 728 individuals aged 18+, of which 20 129 (17.9%) were people who were current tobacco smokers. Of these people who smoke, 1408 were removed from the data sample because they had missing expenditure data, leaving 18 721 people who smoke who had data on their tobacco expenditure for use in the analysis.

### Adjustment for under-reporting of tobacco expenditure in the survey data

From the STS, the self-reported amount spent on tobacco per week by people who smoke was £25.68. Scaled up to annual expenditure on tobacco for the estimated 6.1 million people who smoke in England,^3^ the total is £8.2 billion. However, we expect this to be an underestimate of total expenditure, which requires an upshifting correction. Based on tobacco duty receipts, we estimated the total tobacco expenditure in England to be £11.6 billion. This yielded an upshift factor of 1.4, which has been applied to all estimates of expenditure on tobacco from the STS data in the following analysis. For a detailed breakdown of the calculation of the upshift factor, see online supplemental file 1.

### The value of the smoke-free dividend

**Figure 1:**
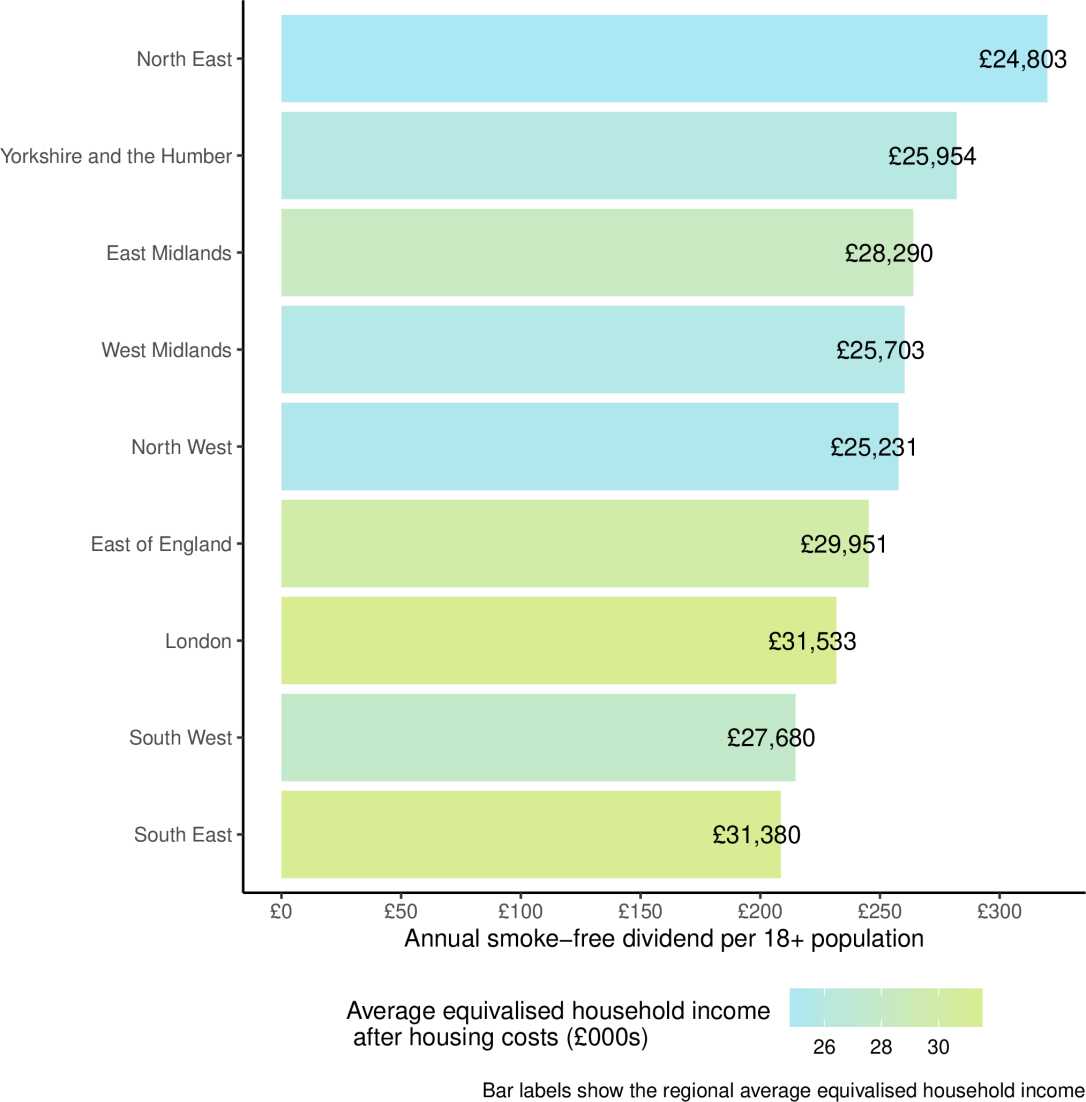
Smoke-free dividend per capita by region. This is the total smoke-free dividend divided by the number of people aged 18+ in each of the nine government office regions in England.

We estimated that the potential smoke-free dividend in England is £10.9 billion (from an estimated total annual expenditure on tobacco of £11.6 billion). Table 1 shows how the value of the smoke-free dividend varies among the nine government office regions in England. In terms of the amount per capita, the dividend would be £246, that is, if everybody stopped smoking, then the economic benefit would be the equivalent of £246 for each adult in England. We estimated that this per capita value of the dividend ranged from £209 in the South East to £320 in the North East (table 1; figure 1). Another way to present the smoke-free dividend is in terms of the economic benefit per person who smokes, which we estimated as £1776, with a range across regions of £1535–£2095 (table 1).

**Table 1:** Regional-level smoke-free dividend

Region	Weekly spend per person who smokes	Average weekly household income	Income spent on tobacco (%)	Total annual spend (million)	Smoking prevalence (%)	People who smoke (n)	Population (18+)	Dividend (million)	Dividend per capita	Dividend per person who smokes
East Midlands	£36.59	£544	6.72	£1078	14.79	566 850	3 832 657	£1012	£264	£1784
East of England	£36.71	£576	6.37	£1279	13.70	669 833	4 889 292	£1199	£245	£1791
London	£36.69	£606	6.05	£1710	12.95	896 639	6 923 853	£1604	£232	£1789
North East	£42.95	£477	9.00	£729	15.27	326 442	2 137 800	£684	£320	£2095
North West	£36.44	£485	7.51	£1588	14.50	837 814	5 778 028	£1489	£258	£1778
South East	£35.28	£603	5.85	£1603	12.12	873 863	7 210 091	£1504	£209	£1721
South West	£31.46	£532	5.91	£1034	13.99	631 799	4 516 076	£970	£215	£1535
West Midlands	£38.03	£494	7.69	£1286	14.03	650 297	4 635 046	£1206	£260	£1855
Yorkshire and the Humber	£36.97	£499	7.41	£1303	15.64	677 670	4 332 928	£1222	£282	£1803
				£11 610		6 131 207	44 255 771	£10 890	£246	£1776

Values used in the calculation of the dividend for each of the nine government office regions in England.

### The smoke-free dividend in relation to the average income of local authority areas

**Figure 2:**
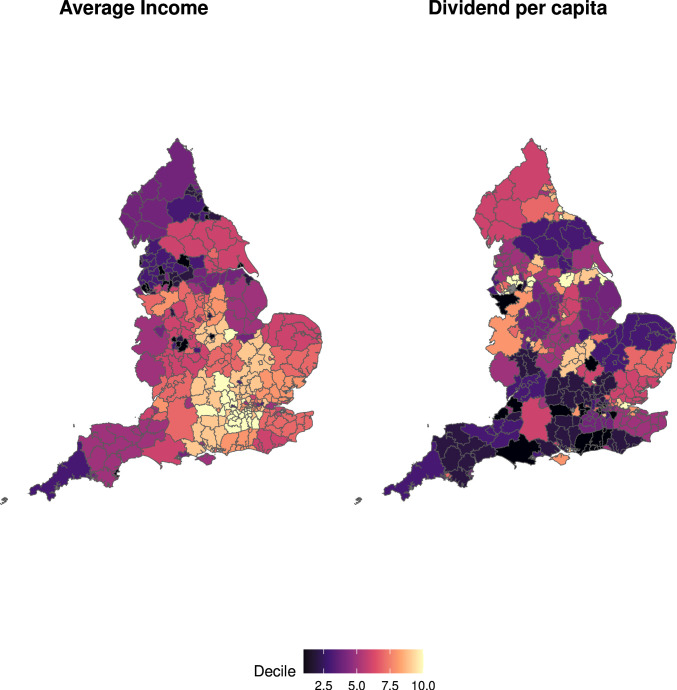
Average income and the smoke-free dividend per capita by local authority. Estimates are shown for 141 of the 151 local authorities in England that had sufficient data to be used in our analysis.

Quantifying variation in the smoke-free dividend across local authority areas in England provides an opportunity to investigate statistically how the smoke-free dividend relates to the average income of local authority areas. Across local authorities, the smoke-free dividend per adult is larger in areas with lower average incomes (correlation coefficient −0.521, 95% CI −0.629 to −0.392; figure 2).

**Figure 3:**
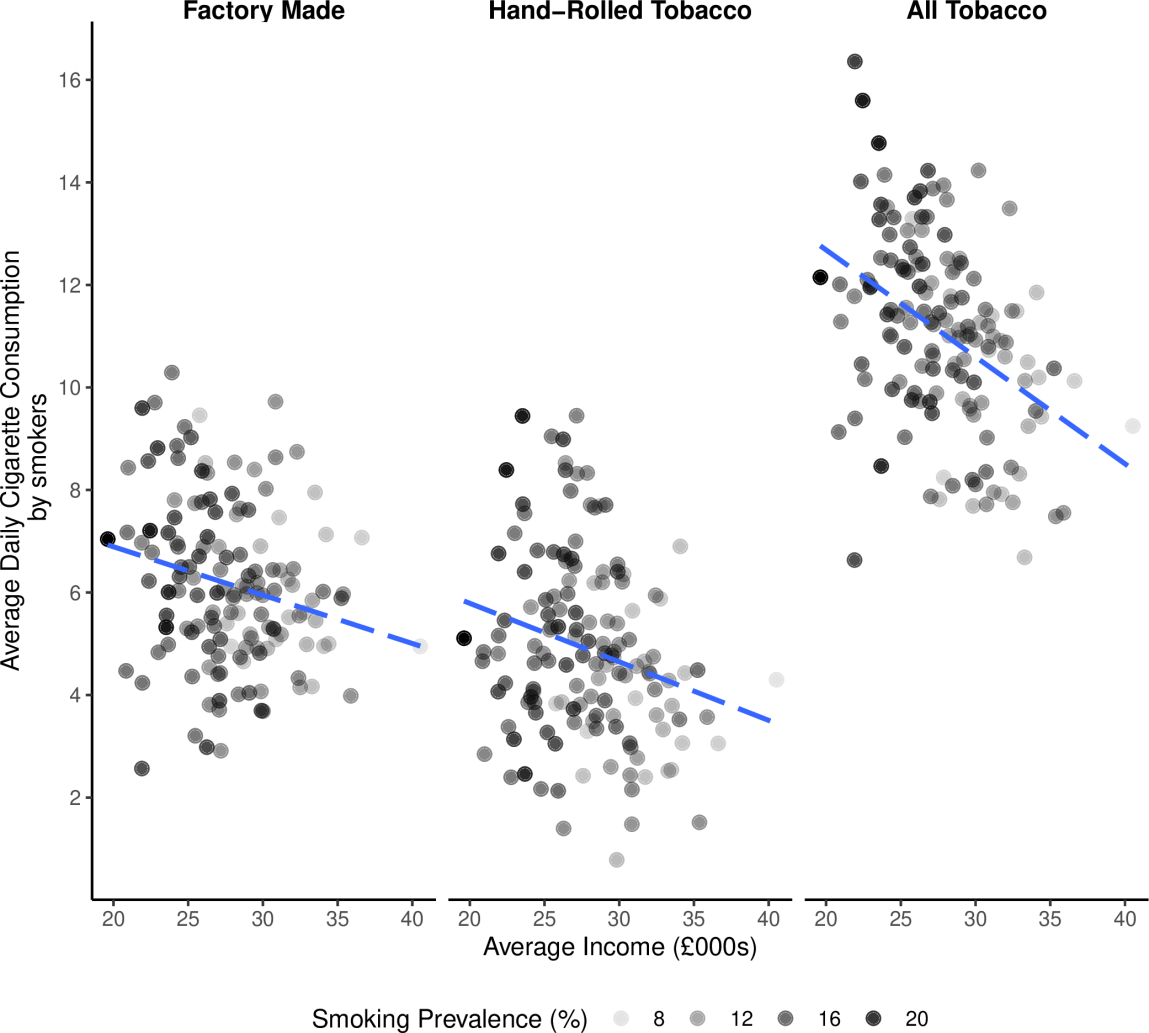
The average number of cigarettes consumed per day in relation to average income. Results are presented separately for the average daily consumption of factory-made cigarettes and hand-rolled tobacco (assuming 0.5 g tobacco per cigarette). Results for ‘all tobacco’, that is, factory-made and hand-rolled tobacco combined, are then presented. In each of the three panels, there are 141 data points corresponding to the local authorities in England with sufficient data to be used in the analysis. The percentage of people who smoke in each local authority is indicated by the grey to black gradient in the points. The lines show the slope of the estimated correlations for each category of tobacco. Plot restricted to local authorities with 10 or more individuals who are smokers in the STS.

**Figure 4:**
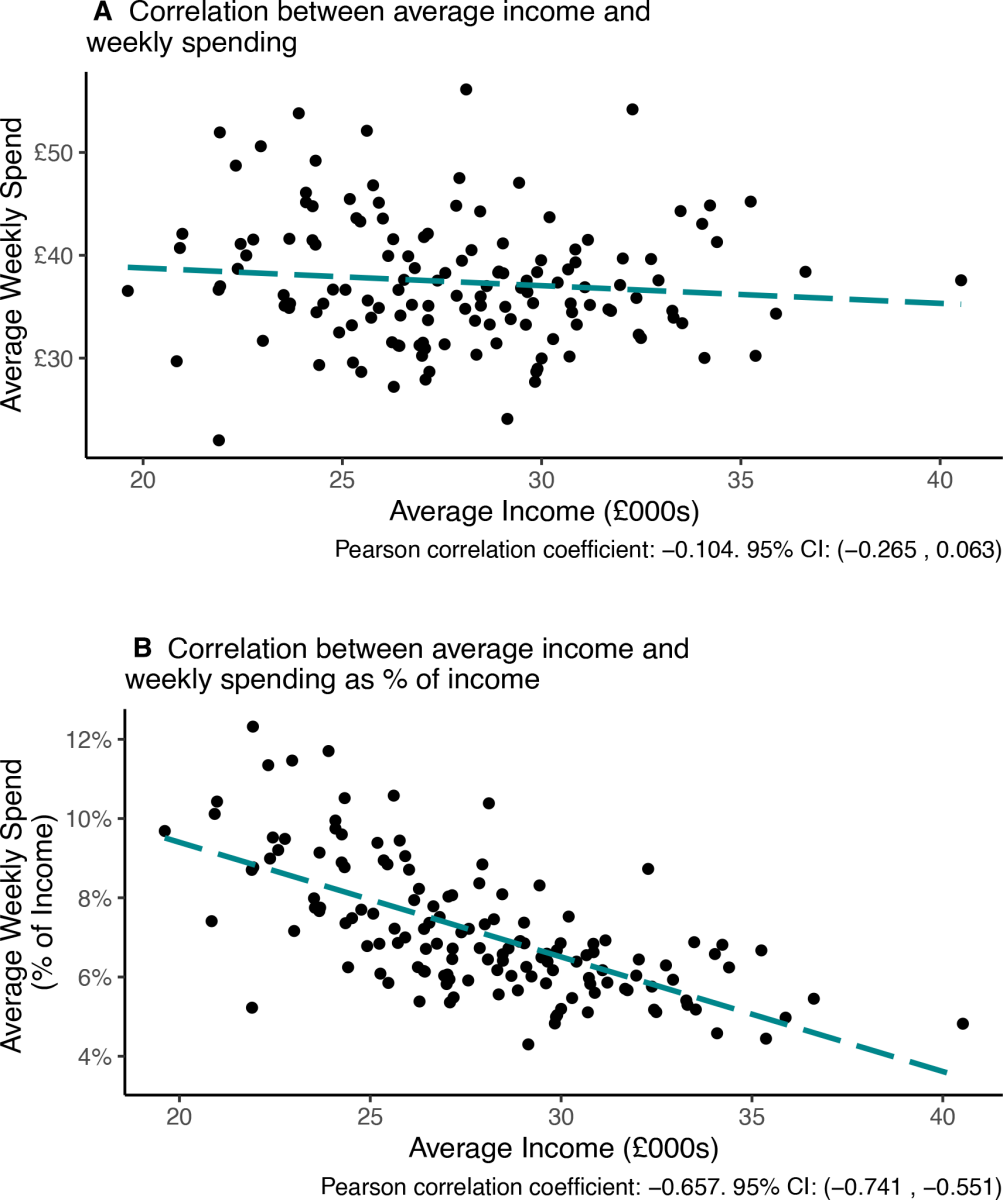
Expenditure on tobacco in relation to average income. Shown in terms of (A) the average weekly expenditure by people who smoke on tobacco, and (B) expenditure on tobacco as a proportion of income. Each data point corresponds to one of the 141 local authorities in England with sufficient data to be used in the analysis. The line shows the slope of the estimated correlation. Plot restricted to local authorities with 10 or more individuals who are smokers in the STS.

To understand the reasons why the smoke-free dividend is higher in lower income areas, it is helpful to investigate the relationships between local area income, smoking behaviour and the amount spent on tobacco. Lower income local authorities have a higher percentage of adults who smoke, and also the people who smoke tend to smoke a larger number of cigarettes per day (−0.413, 95% CI −0.541 to −0.265; figure 3). However, we did not find a statistically significant association between the average income of local authority areas and the amount that people who smoke spent on tobacco (−0.104, 95% CI −0.265 to 0.063; figure 4A). That people who smoke in lower income areas smoke more cigarettes per day, but do not spend more on tobacco indicates that they are buying cheaper tobacco. The conclusion is that smoke-free dividends are higher in lower income areas because these areas have higher percentages of people who smoke, and not because individuals who smoke are spending more on tobacco.

**Figure 5:**
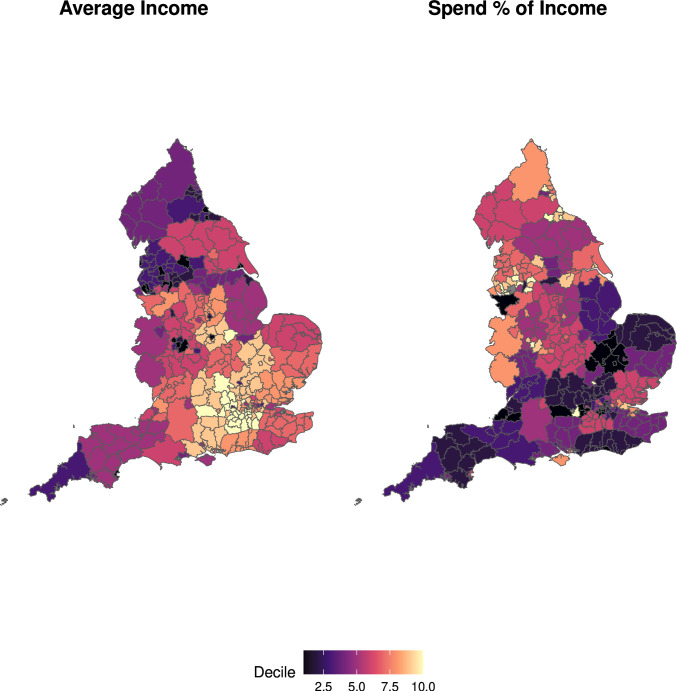
Average income and the financial burden of tobacco by local authority. Financial burden is defined as expenditure on tobacco as a proportion of income.

Finally, we investigated variation in the smoke-free dividend according to the proportion of their income that people who smoke spent on tobacco. This relationship is useful to know because the relative value of the dividend would be higher for people who smoke who spend more of their income on tobacco. Spending on tobacco was higher as a proportion of income for people who smoke who live in lower income areas (−0.657, 95% CI −0.741 to −0.551; figure 4B). Thus, the highest smoke-free dividends would be received by smokers who quit smoking and who previously spent the highest proportion of their income on tobacco (figure 5).

## Discussion

The potential smoke-free dividend for the economy in England is £10.9 billion, which equates to £246 per adult and £1776 per person who smokes. This value is larger than the previous estimate for England of £7 billion,^1^ but that earlier estimate did not account for under-reporting of tobacco consumption in survey data or for expenditure on illicit tobacco. We found that the smoke-free dividend would be highest in lower income areas, where rates of smoking are higher and where expenditure on tobacco constitutes a higher proportion of income. However, our analysis does not account for the multiple sociodemographic influences on smoking,^20^ and in some communities the financial burden of smoking is larger than we have estimated, and so the consequent smoke-free dividend would also be larger.

UK households are currently under particular financial pressures due to the impact of inflation on energy, fuel and food prices.^8^ These increases in financial outgoings have a disproportionate impact on low-income households, who consequently spend a higher proportion of their income on essential goods; half of households in the UK reported spending less on non-essentials in early 2022.^8^ In households who spend money on tobacco, particularly low-income households, the proportion of income spent on tobacco is significant. Several studies have demonstrated that tobacco expenditure ‘crowds out’ expenditure on household essentials such as housing, food and education.^21^,^26^ Previous research has demonstrated the existence of smoking-induced deprivation, whereby households forgo essentials due to expenditure on tobacco.^27^ The prevalence of smoking-induced deprivation is, unsurprisingly, higher in low-income households, where tobacco products can be seen as a ‘protected purchase’.^28^ Quitting smoking would free up disposable income that could be redirected to other expenditures, to pay-off debts or saved. It is important to note that there are ethical challenges associated with categorising tobacco expenditure as ‘unnecessary expenditure’ which could be redirected to ‘better’ spending decisions. It might also be argued that our study does not capture the value of smoking to people who smoke. However, it is also important to note that there is no safe level of smoking,^29^ and most people who smoke in the UK want to quit and have made several attempts to do so.^30^

Our study should be seen as providing information to motivate further policy action to reduce rates of smoking. The findings of our study put a value on the potential financial benefit to local economies of making smoking obsolete. This financial benefit is in addition to the direct health benefits to people who smoke of stopping smoking, and the knock-on economic effects of those health benefits, for example, better health can lead to increased work productivity and reduced healthcare costs. For England, the government has a target to make the country smoke free, which means that it aims to reduce the percentage of adults who smoke to below 5% by 2030.^9^ However, modelling indicates that in England, people living in the most deprived socioeconomic conditions are likely to lag far behind in achieving this target, with the percentage of people who smoke in the most deprived areas not likely to fall below 5% until the mid-2040s.^1^ Future tobacco control policy will therefore need to be more ambitious and focus on the most disadvantaged communities.^1 31^ Our findings show the potential economic benefits that those disadvantaged communities themselves might gain from this ambitious policy action. However, it should be noted that making smoking obsolete would also bring financial costs through the loss of tax revenue to government, which can also have benefits to local economies. From a whole-economy perspective, the actual smoke-free dividend would be the net of all these economic impacts. The smoke-free dividend has a greater local impact, as local dividends are higher in areas with greater smoking prevalence, whereas there is no guarantee that tax revenue from tobacco is redistributed in proportion to the geographical distribution of smoking prevalence rates.

The financial burden of smoking to households in England has been investigated in earlier studies.^4 6^ However, our study is the first to provide a comprehensive estimate of the potential smoke-free dividend to local economies and to provide detailed information on geographical variations in this dividend. Our approach is comprehensive because it adjusts for under-reporting of tobacco consumption in survey data and includes an estimate of expenditure on illicit tobacco. Illicit tobacco undermines the effectiveness of interventions to reduce smoking prevalence by giving people who smoke access to a cheap alternative to duty-paid tobacco. While the estimate of expenditure on illicit tobacco is approximate and varies depending on enforcement action, including it in estimates of the potential smoke-free dividend should give a better indication of the true magnitude of the dividend. We have not considered the reduced spending on healthcare in the longer term that would result from stopping smoking, as in the English context individuals do not have to personally fund their own healthcare costs, and so these savings would go back to the National Health Service as a national rather than local economic impact. In other countries, where individuals have to pay for their own healthcare, these savings would accrue to those individuals who would then be able to spend that money locally, adding to the smoke-free dividend.

A limitation of our study is that it cannot provide information on what would be done with the money released to people who quit smoking if it were no longer spent on tobacco. This means that when we report our estimate of the potential smoke-free dividend to local economies, we are assuming that all the money that people who quit smoking get back would actually be spent locally on goods and services. That is a strong assumption, which is made for illustrative purposes in the knowledge that not all of that money would find its way into the economies local to where the people who smoke live. Research in the USA has suggested that following smoking cessation households spend less overall, suggesting that they might save the money previously spent on tobacco or use it to pay debts.^32^ Households were found to spend less on goods and in areas that were linked to their previous smoking, such as on alcohol and entertainment. The study also identified reduced spending on food at home following smoking cessation, although this was not sustained in the long run. Investigation of how resources previously spent on tobacco are reallocated is needed to improve understanding of the economic impact of stopping smoking. In particular, this study has not considered e-cigarettes; people who quit smoking may take up vaping and therefore still be spending money on addictive commodities. However, irrespective of how that money is spent, it is unlikely to be on a substance as harmful as tobacco. Finally, we assume that individuals include both legal and illicit spending in the total weekly spending they report, and also that the division of spending between these two sources is the same in all local authorities which may not be the case.

While our findings relate only to a specific time period in England, our study presents a method for quantifying the potential economic benefits to any nation or subnational region of becoming smoke free. Regions might differ in how they describe their ambitions to reduce rates of tobacco smoking, for example, they might use ‘smoke-free’, ‘tobacco-free’ or ‘making smoking obsolete’, and the targets associated with these ambitions might vary in terms of the target percentage of people who smoke and the year of reaching this target. However, in many countries tobacco use is most common among people in poorer socioeconomic groups, and therefore our findings are likely to be relevant beyond England. Our methods can also be generalised to produce comparable calculations for other countries or regions where there are data on tobacco smoking prevalence and expenditure by local area, and an estimate of the proportion of tobacco expenditure that is retained by local retailers. In particular, the smoke-free dividend will likely be much higher in economies that have higher smoking rates than England. Across regions, it will be relevant for policy action to show that becoming smoke free can relieve some of the financial burden on people who live in the most deprived sections of the population, and this need has now increased in all countries in the context of current worldwide inflationary pressures.

In conclusion, we have shown the potential scale of the benefits to local economies of making smoking obsolete, and that these benefits are likely to be greatest for areas with the lowest average incomes. This means that investment to reduce smoking rates is likely to help reduce geographical economic inequalities.

## Supplementary material

10.1136/tc-2023-058264 online supplemental file 1

10.1136/tc-2023-058264 online supplemental file 2

## Data Availability

Data are available in a public, open access repository.
