# Supplementary material for: Potential smoke-free dividend across local areas in England: a cross-sectional analysis
Source: Tob Control. 2024 Mar 20;34(4):e058264. doi: 10.1136/tc-2023-058264 (PMC12322449; doi:10.1136/tc-2023-058264)
Supplement: online supplemental file 1 [file tc-34-4-s001.pdf]

# Supplementary Information for “The potential smoke-free dividend across local areas in England: A cross-sectional analysis”

## Supplement 1 – Calculating the Upshift

Under-reporting of expenditures in the Smoking Toolkit Study (STS) [1] means estimates of the smoke-free dividend calculated at local authority level will be too small. We describe here in detail the methodology and assumptions used to identify the extent to which the survey data under-estimates expenditures on tobacco, and to construct the upshift factor used in the analysis to adjust under-reported figures. A step-by-step breakdown of the upshift factor calculation is presented in Table S1.

## Adjustment for under-reporting of tobacco expenditure in the survey data

From the STS, the self-reported amount that people who smoke spent on tobacco was £25.68 per week. When this is scaled up to annual expenditure on tobacco for the estimated 6.1 million people who smoke in England [2], the total is £8.2 billion. However, we expect this to be an under-estimate of total expenditure, which requires an upshifting correction. The reference that we used to estimate the upshift factor was the total tobacco duty (excluding Value Added Tax) collected by the UK government, which was £9.2 billion in the tax year 2018/19 [3]. To obtain the value for England from this whole-of-UK value, we scaled it downwards based on a reported 76.6% of tobacco duty receipts in 2018/19 coming from England [4].

Total expenditure was calculated from duty receipts by estimating the percentage of the retail price for each product that consisted of excise duty. The price figure used for factory-made cigarettes was an estimate of the weighted average price of all brands [5]. For HRT, the price was calculated as the average price from ONS price quote data, which underpins the UK consumer price inflation series [6]. Calculations were made on the assumption of 0.5g tobacco per hand-rolled cigarette. The prices for cigarettes and HRT respectively were £8.08 per pack of 20 cigarettes [5] and £45.52 per 100g [6]. We calculated total duty per pack (20 cigarettes, 100g of HRT) based on the duty rates in place in December 2018 [3] of £228.29 per 1,000 cigarettes plus ad-valorem tax of 16.5% of the retail price, and £234.65 per kilogram of HRT. These figures were adjusted to calculate a duty per pack for cigarettes and HRT, and this figure as a percentage of the retail pack price was used to scale total duty receipts by HMRC up to a total expenditure figure.

## Illicit tobacco expenditure

As some consumption of tobacco products is from illicit sources, which we assumed is captured in the spending reported in the STS, we also estimated expenditure on illicit

tobacco. Using government estimates from the 2018/19 tax year of the volumes of illicit cigarettes and HRT [7], expenditures on illicit sources of consumption were calculated as the volume of illicit consumption multiplied by the estimated average price of illicit tobacco products. The average cost of illicit sources of both HRT and cigarettes have been estimated at approximately half of the average legal price [8]. We therefore adjusted the prices for legally sourced tobacco in December 2018 by 50% and applied these illicit prices to the estimated volumes of illicit consumption to estimate the figure for expenditure on illicit tobacco. This expenditure was added to the total expenditure on legal sources of tobacco to obtain total expenditure on legal and illicit sources of tobacco.

The upshift factor is the ratio of the reference value for total tobacco expenditure in England (£11.6 billion) to the equivalent figure implied by the self-reported data on tobacco expenditure in the STS (£8.2 billion). This yields an upshift factor of 1.417 which has been applied to all estimates of individual expenditure on tobacco derived from the STS in the analysis.

**Table S1. Breakdown of the Upshift Calculation**

| Factory-Made Cigarettes | | | Hand-Rolled Tobacco | | | Totals (£bn) |
|---|---|---|---|---|---|---|
| **HMRC data estimate** | | | | | | |
| (1) Total duty receipts (£bn) | | £5.935 | (1) Total duty receipts (£bn) | | £1.106 | |
| (2) Pack Price (£ per 20 cigs) | | £8.08 | | | | |
| (3) Ad-Valorem tax rate | | 16.50% | | | | |
| (4) Ad-Valorem tax (£ per pack) | (2) * (3) | £1.33 | (2) Price per 100g (Dec 2018) | | £45.52 | |
| (5) Specific duty (£ per 1000 cigs) | | £228.29 | (3) Specific duty (£ per kg) | | £234.65 | |
| (6) Specific duty (£ per pack of 20) | (5) / 50 | £4.57 | (4) Specific duty (£ per 100g) | (3) / 10 | £23.46 | |
| (7) Total excise (£ per pack) | (4) + (6) | £5.90 | | | | |
| (8) Total excise % of price | (7) / (2) | 72.99% | (5) Total excise % of price | (4) / (2) | 51.55% | |
| (9) Total legal cigarette spend (£bn) | (1) / (8) | £8.131 | (6) Total legal HRT spend (£bn) | (1) / (5) | £2.146 | **£10.277** |
| (10) Total illicit cigarette spend (£bn) | | £0.506 | (7) Total illicit HRT spend (£bn) | | £0.819 | **£1.325** |
| | | | | | | **£11.602** |
| **Smoking Toolkit Study estimate** | | | | | | |
| Total grossed-up expenditure | | | | | | **£8.187** |
| **Upshift Factor:** | 1.417 | | | | | |

## References

[1] Fidler JA, Shahab L, West O*, et al.* The Smoking Toolkit Study: A national study of smoking and smoking cessation in England. *BMC Public Health* 2011;**11**:479 https://doi.org/10.1186/1471-2458-11-479

[2] Office for Health Improvement and Disparities. Local Tobacco Control Profiles. 2023 https://fingertips.phe.org.uk/profile/tobacco-control

[3] HM Revenue and Customs. Tobacco bulletin. 2023 https://www.gov.uk/government/statistics/tobacco-bulletin

[4] HM Revenue and Customs. A disaggregation of HMRC tax receipts between England, Wales, Scotland & Northern Ireland. 2019 https://assets.publishing.service.gov.uk/government/uploads/system/uploads/attachment_data/file/853113/Disaggregated_tax_and_NICs_receipts_-_information_and_analysis.pdf

[5] European Commission. Indirect taxes - Excise duty - Manufactured tobacco. 2020 https://ec.europa.eu/taxation_customs/tedb/taxDetails.html?id=4100/1514764800

[6] Office for National Statistics. Consumer price inflation item indices and price quotes. 2023 https://www.ons.gov.uk/economy/inflationandpriceindices/datasets/consumerpriceindicescpiandretailpricesindexrpiitemindicesandpricequotes

[7] HM Revenue and Customs. Measuring tax gaps 2021 edition - tax gap estimates for 2019 to 2020. 2021 https://webarchive.nationalarchives.gov.uk/ukgwa/20220614163810/https://www.gov.uk/government/statistics/measuring-tax-gaps

[8] ASH Scotland. Calculating the cost of smoking. 2021 https://www.ashscotland.org.uk/media/850413/28-calculating-the-cost-of-smoking-june-2021.pdf
