# Supplementary material for: Potential smoke-free dividend across local areas in England: a cross-sectional analysis
Source: Tob Control. 2024 Mar 20;34(4):e058264. doi: 10.1136/tc-2023-058264 (PMC12322449; doi:10.1136/tc-2023-058264)
Supplement: online supplemental file 2 [file tc-34-4-s002.pdf]

Supplementary Information for "The potential smoke-free dividend across local areas in England: A cross-sectional analysis"

Supplement 2 – Sources for parameters and data

The tobacco expenditure data is sourced from the Smoking Toolkit Study (STS) [1] (https://smokinginengland.info). To run the analysis the user must provide their own version of the STS data.

All other analysis data are publicly available and detailed below, along with their sources, in Table S2.

For those interested in replicating the analysis, the code is openly available on the GitHub open source code repository [2]. This code relies on additional code functions and formatted data, openly shared in the R package "smkfreediv", also accessible on GitHub [3]. The data within this R package corresponds to the sources listed in Supplement 2, and has been formatted to integrate with the reproducible analysis code.

Table S2. Sources for parameters and data.

Data	Source	Description
Consumer Price Index (Tobacco)	Office for National Statistics (ONS) [4]	Consumer price inflation time series for tobacco products.<br><br>URL: CPI INDEX 02.2 : TOBACCO 2015=100 - Office for National Statistics (ons.gov.uk)
Annual household income	Office for National Statistics (ONS) [5]	Estimates of annual household income for Middle layer Super Output Areas (MSOAs) in England and Wales.<br><br>Data used were net equivalised household income after housing costs using the OECD equivalence scale [6]. The OECD equivalence scale gives a weight of 1 to the income of the first household member, 0.7 to additional adults, and 0.5 to any children.<br><br>URL: Income estimates for small areas, England and Wales - Office for National Statistics (ons.gov.uk)
Local Tobacco Control Profiles	Office for Health Improvement and Disparities (OHID) [7]	Estimates of the number and percentage of people who are current regular tobacco smokers at local authority level.<br><br>URL: Local Tobacco Control Profiles - OHID (phe.org.uk)
Illicit tobacco consumption	HM Revenue and Customs (HMRC) [8]	Measuring tax gaps data. The tax gap is the difference between the amount of tax that should, in theory, be paid to HMRC, and what is actually paid.<br><br>URL: Measuring tax gaps tables - GOV.UK (www.gov.uk)
Price – Factory-made cigarettes	European Commission [9]	Database: European Commission, taxation, and customs data.<br><br>URL: Taxes in Europe Database v3 (europa.eu)
Price – Handrolled tobacco	Office for National Statistics (ONS) [10]	The price of handrolled tobacco (HRT) is taken as the average of the individual prices for HRT in the price quotes data which underpin the consumer price index.<br><br>URL: Consumer price inflation item indices and price quotes - Office for National Statistics
Price – Illicit tobacco	ASH Scotland [11]	Report: Calculating the cost of smoking<br><br>URL: https://www.ashscotland.org.uk/media/850413/28-calculating-the-cost-of-smoking-june-2021.pdf
Tobacco duty rates & receipts	HM Revenue and Customs (HMRC) [12]	Tobacco bulletin – monthly statistics on duty rates, receipts and clearances for cigarettes and other tobacco products.<br><br>URL: Tobacco Bulletin - GOV.UK (www.gov.uk)<br><br>To obtain the value of duty receipts for England from the whole-of-UK value, we scaled it downwards based on the estimated percentage of tobacco duty receipts coming from England [13].

References

[1] Fidler JA, Shahab L, West O*, et al.* The Smoking Toolkit Study: A national study of smoking and smoking cessation in England. *BMC Public Health* 2011;**11**:479 https://doi.org/10.1186/1471-2458-11-479

[2] Morris D, Gillespie D, Dockrell M*, et al.* Reproducible code for: The potential smoke-free dividend across local areas in England: A cross-sectional analysis. Github open access code repository 2023 https://github.com/STAPM/smoke-free-dividend https://doi.org/10.17605/OSF.IO/VZMP7

[3] Morris D, Gillespie D, Dockrell M*, et al.* smkfreediv: An R Package for Smoke-Free Dividend Calculations. Version 1.6.3. Github open access code repository 2023 https://github.com/STAPM/smkfreediv

[4] Office for National Statistics. Consumer price inflation time series. 2023 https://www.ons.gov.uk/economy/inflationandpriceindices/datasets/consumerpriceindices

[5] Office for National Statistics. Income estimates for small areas, England and Wales. 2023 https://www.ons.gov.uk/employmentandlabourmarket/peopleinwork/earningsandworkinghours/datasets/smallareaincomeestimatesformiddlelayersuperoutputareasenglandandwales

[6] Office for National Statistics. Income estimates for small areas technical report: Financial year ending 2014. 2016 https://www.ons.gov.uk/employmentandlabourmarket/peopleinwork/earningsandworkinghours/methodologies/smallareaincomeestimatesmodelbasedestimatesofthemeanhouseholdweeklyincomeformiddlelayersuperoutputareas201314technicalreport

[7] Office for Health Improvement and Disparities. Local Tobacco Control Profiles. 2023 https://fingertips.phe.org.uk/profile/tobacco-control

[8] HM Revenue and Customs. Measuring tax gaps 2021 edition - tax gap estimates for 2019 to 2020. 2021 https://webarchive.nationalarchives.gov.uk/ukgwa/20220614163810/https://www.gov.uk/government/statistics/measuring-tax-gaps

[9] European Commission. Indirect taxes - Excise duty - Manufactured tobacco. 2020 https://ec.europa.eu/taxation_customs/tedb/taxDetails.html?id=4100/1514764800

[10] Office for National Statistics. Consumer price inflation item indices and price quotes. 2023 https://www.ons.gov.uk/economy/inflationandpriceindices/datasets/consumerpriceindicescpiandretailpricesindexrpiitemindicesandpricequotes

[11] ASH Scotland. Calculating the cost of smoking. 2021 https://www.ashscotland.org.uk/media/850413/28-calculating-the-cost-of-smoking-june-2021.pdf

[12] HM Revenue and Customs. Tobacco bulletin. 2023 https://www.gov.uk/government/statistics/tobacco-bulletin

[13] HM Revenue and Customs. A disaggregation of HMRC tax receipts between England, Wales, Scotland & Northern Ireland. 2019 https://assets.publishing.service.gov.uk/government/uploads/system/uploads/attachment_data/file/853113/Disaggregated_tax_and_NICs_receipts_-_information_and_analysis.pdf
